# The Common Accidents of Every-Day Life

**Published:** 1888-03-03

**Authors:** 


### The Common Accidents of Every-day Life.

By a Surgeon.

### Fractures and Dislocations.

Fractures have been divided into—

*Simple*, where the bone is merely broken across transversely or obliquely at one point.

*Comminuted*, where the bone is broken into fragments.

*Compound*, where there exists also an external wound communicating with the bone at the point of fracture.

*Compound comminuted*, where the wound communicates with the fractured bone, the bone itself being shattered into fragments.

*Greenstick*, which almost always occurs in the young, before the bone is thoroughly ossified. In such cases the bone bends, and breaks partially in the convexity. It generally occurs in the forearm or collar-bone.

The exciting causes of fractures and dislocations are the same, viz., mechanical violence and muscular action. The former acts either directly or indirectly. For instance, I was the subject of a dislocation of the left humerus by direct violence. Whilst playing football I charged, and was charged at by one of the opposite side, and received the full force of the encounter on the left shoulder. My opponent fell, but I sustained a dislocation, the head of the bone being driven into the axilla, or armpit. If a person fell upon the knee, and dislocated the head of the thigh-bone or femur, this would be an example of violence acting indirectly. A good example of dislocation through muscular action is that of the lower jaw in the act of yawning, which sometimes occurs. Yawning is infectious, as anyone may test for himself. A good story is told—for the truth of which, however, I cannot vouch—of a certain Scotch professor having his lower jaw dislocated in this way. He had previously suffered from the accident, and was therefore a good subject in which to perpetuate the mischief which ensued. Some of his students commenced a series of yawns, the infection gradually spreading over the class until it reached the professor himself, who in turn began to yawn very vigorously—too much so for his own comfort, for he managed once more to dislocate his jaw, doubtless to the amusement and satisfaction of some of his pupils.

As to the length of the limb, it may be either shortened or lengthened in dislocation; in fracture, with one or two exceptions, it is *always* shortened. So that you see *the* great differences between the two accidents are crepitus in fracture, and its absence in dislocation; immobility of the limb in dislocation, and its opposite in fracture.

The exciting causes of fracture are as has been said of two kinds—mechanical violence and muscular action; the former is the more frequent, and may be either *direct* or *indirect*. When a person receives a kick, as from a clog—a very common method of producing a fracture in Lancashire—upon the head or ribs, it may cause one *directly*; but if a person were to fall from a height, and alight upon his feet and fracture the leg or the thigh, this would be an instance of *indirect* violence.

Fractures produced by muscular action have occurred in persons of uncommon vigour; *e.g.*, a well-developed and well-known cricketer fractured his humerus by throwing in the ball to the wicket-keeper with extraordinary force; a negro preacher broke the same bone when gesticulating violently, and a dentist was the victim of a similar accident on attempting to draw a tooth. The patella, or knee-pan, is often fractured in the same way. I knew a case in which a clown in a circus fractured this bone in turning a somersault; and an athlete broke his thigh in suddenly twisting round whilst playing skittles. It has been remarked by some that fractures are more common in winter than in summer, the reasons being that then there is greater liability to falls from the slippery state of the ground, and also from the bones being more brittle at this time.

*(To be continued.)*

